# Review of withdrawal catatonia: what does this reveal about clozapine?

**DOI:** 10.1038/s41398-018-0192-9

**Published:** 2018-07-31

**Authors:** Matthew Lander, Tarun Bastiampillai, Jitender Sareen

**Affiliations:** 1 Department of Psychiatry, University of Manitoba, Winnipeg, Canada; 2 Discipline of Psychiatry, School of Medicine, Flinders University, Adelaide, Australia; 3 South Australian Health and Medical Research Institute, Adelaide, Australia; 4 Departments of Psychiatry, Psychology, and Community Health Sciences, University of Manitoba, Winnipeg, Canada

## Abstract

Withdrawal symptoms are common upon discontinuation of psychiatric medications. Catatonia, a neuropsychiatric condition proposed to be associated with gamma-aminobutyric acid (GABA) hypoactivity due to its robust response to benzodiazepines, has been described as a withdrawal syndrome in case reports but is not a well-recognized phenomenon. The authors undertook a review of withdrawal catatonia with an aim to understand its presentation as well as the medications and psychoactive substances it is associated with. The review identified 55 cases of withdrawal catatonia, the majority of which occurred upon discontinuation of benzodiazepines (24 cases) and discontinuation of clozapine (20 cases). No other antipsychotic medications were identified as having been associated with the onset of a catatonic episode within 2 weeks following their discontinuation. Increasing GABA activity and resultant GABA receptor adaptations with prolonged use is postulated as a shared pharmacological mechanism between clozapine and benzodiazepines that underlie their association with withdrawal catatonia. The existing evidence for clozapine’s activity on the GABA system is reviewed. The clinical presentations of benzodiazepine withdrawal catatonia and clozapine withdrawal catatonia appear to differ and reasons for this are explored. One reason is that benzodiazepines act directly on GABA_A_ receptors as allosteric agonists, while clozapine has more complex and indirect interactions, primarily through effects on receptors located on GABA interneurons. Another possible reason for the difference in clinical presentation is that clozapine withdrawal catatonia may also involve receptor adaptations in non-GABA receptors such as dopamine and acetylcholine. The findings from our review have implications for the treatment of withdrawal catatonia, and treatment recommendations are provided. Further research understanding the uniqueness of clozapine withdrawal catatonia among antipsychotic medication may give some insight as to clozapine’s differential mechanism of action.

## Introduction

Catatonia is a serious neuropsychiatric condition that has been associated with a wide range of psychiatric, medical, neurologic, and drug-induced conditions. Catatonia has been described as having two subtypes, a stuporous form that includes signs and symptoms of mutism, rigidity, immobility, negativism, posturing, and catalepsy, and an excited form that includes excitement, aggression, and impulsivity. Additionally, a severe and life-threatening form that is associated with autonomic instability and fever is known as malignant catatonia.

Benzodiazepines are the first-line treatment for catatonia regardless of the underlying cause^1^ with high treatment response rates^2–5^. Benzodiazepines exert their effects on GABA_A_ receptors, which are classified as ligand-gated ion channels. When GABA binds to its binding site on the GABA_A_ receptor, it increases the frequency of opening of the receptor chloride channel, allowing more chloride to pass through, resulting in an inhibitory effect. The flow of ions through the channel also depends on the concentration gradient of the ions and the membrane potential of the cell. Benzodiazepines are classified as a positive allosteric modulator, as they bind to a separate site on the GABA_A_ receptor and amplify the effect of GABA on the GABA_A_ receptor. The presence of a benzodiazepine at a GABA_A_ receptor increases the frequency of opening of the chloride channel more than when GABA alone is present. Benzodiazepines have no activity on their own, and thus require the presence of GABA at the GABA_A_ receptor to exert their effect^6^. The well-established efficacy of benzodiazepines in the treatment of catatonia implicates GABA hypoactivity in the pathophysiology of catatonia.

ECT has also been established as being highly effective for catatonia^2^ and is suggested in benzodiazepine-resistant cases and in cases with life-threatening features^1^. ECT has broad effects on the central nervous system including increasing serum GABA levels and GABA_B_ activity^7,8^. This lends further support to a GABA deficit model of catatonia.

The use of typical antipsychotics is discouraged in patients presenting with catatonic features due to inefficacy and the potential risk of worsening symptoms of catatonia. Furthermore, administration of antipsychotic medications can cause a catatonic episode. This is known as neuroleptic-induced catatonia and has been reported with both typical and atypical antipsychotics^9–13^. Neuroleptic-induced catatonia has overlapping symptoms with neuroleptic malignant syndrome including autonomic abnormalities. As such, neuroleptic-induced catatonia has been proposed to be a mild variant of neuroleptic malignant syndrome^11^, a syndrome believed to be due to dopamine blockade. The clinical findings that catatonia can be precipitated or worsened by the administration of dopamine blocking agents and that neuroleptic-induced catatonia shares similarities with neuroleptic malignant syndrome suggest the role of dopamine hypoactivity in the pathophysiology of catatonia.

An evolutionary model of catatonia as a primitive response to fear that is triggered by extreme physical or psychological stress has also been proposed^14^. In this model, catatonia may be a form of the animal defense strategy of tonic immobility, which is the sudden onset of prolonged stillness or “freezing” when an animal is exposed to a threatening stimulus^15^. In the animal world, tonic immobility may increase the chances of survival by helping avoid predators that are triggered by movement. Catatonia and tonic immobility share many features including immobility, posturing, stupor, waxy flexibility, mutism, and abrupt onset^16^. Observations that support a model of catatonia as a fear response are high rates of fear during a catatonic episode reported by patients after the episode resolves^3^, the frequent presence of symptoms of autonomic arousal during a catatonic episode^17^, and benzodiazepines, which are an effective treatment for catatonia, having anxiolytic properties.

While clozapine is well-established to have antipsychotic properties, it’s effect on catatonia is not fully known. There is evidence that clozapine has a unique feature among antipsychotics in improving signs and symptoms of catatonia rather than causing or worsening catatonia. This has been reported in cases of catatonia secondary to both psychiatric^18–25^ and neurologic^26^ conditions. In most of these cases, clozapine was used after unsuccessful trials of the established anticationic agents benzodiazepines and ECT, supporting its potential efficacy in treatment-resistant cases of catatonia. Some of these cases involve catatonic presentations in individuals with schizophrenia, and therefore clozapine may have exerted its superior effect through treatment of the underlying psychotic illness. Clozapine was also however found to be effective in treating catatonia related to nonpsychotic illnesses in two cases of major depressive disorder^22^ and one case of traumatic brain injury^26^. This limited evidence suggests that clozapine potentially may have some primary anticatatonic effects beyond its well-established antipsychotic properties.

Many medications used in psychiatry are associated with physical and psychological withdrawal symptoms that occur upon medication discontinuation. These withdrawal symptoms are generally characterized as rebound effects in the same physiological systems that were acted upon by the medication^27^. Benzodiazepines, for instance, are associated with multiple patterns of withdrawal symptoms, the most common being rebound anxiety and insomnia that occurs 1−4 days after discontinuation^27^. A less recognized withdrawal syndrome that has been associated with benzodiazepine discontinuation is withdrawal catatonia^28^. Withdrawal catatonia has also been associated with clozapine discontinuation, as previously reported by the authors^29^ and recently reviewed by Bilbily et al.^30^. Knowing that benzodiazepines and clozapine are both treatments for catatonia, it is possible that these cases are indicative of a phenomenon of “rebound” catatonia occurring when discontinuing medications used to treat catatonia. This would be analogous to the clinical presentations seen following the discontinuation of benzodiazepines (rebound anxiety) and antipsychotics (rebound psychosis). The aim of our review was to expand on previous reviews of sedative^28^ and clozapine^30^ withdrawal catatonia by ascertaining all medications and psychoactive substances that have been associated with “withdrawal catatonia”. Furthermore, we were interested in determining potentially common pharmacological characteristics that may underlie this shared occurrence.

## Methods

A review of several databases (Pubmed, Medline, and Embase) was conducted using the key terms “withdrawal catatonia”, “discontinuation catatonia”, and “rebound catatonia”. The electronic search was supplemented by hand searching the bibliographies of papers selected from the electronic search and checking references of review articles. Two reviewers (M.L. and T.B.) independently conducted the searches, paper selection, and data extraction.

We defined a withdrawal event as having occurred in 14 days or less following discontinuation of a medication or other psychoactive substance. An event occurring at a longer time interval from the time of discontinuation would have too great a likelihood of being due to an alternate mechanism (i.e. relapse of underlying illness) to be identified as a withdrawal event. A diagnosis of catatonia was included if a diagnosis was made in the published case. The confidence of the diagnosis being catatonia as opposed to an alternative phenomenon was assessed in each case by the two reviewers based on two factors: (1) if a validated catatonia rating scale was used to make the diagnosis and (2) if DSM-V criteria for a diagnosis of catatonia was met. The DSM-V requires 3 or more of a possible 12 symptoms for a diagnosis of catatonia^31^.

We included papers published in full that reported on the occurrence of a catatonic episode occurring in relation to the withdrawal of any medication or other psychoactive substance. We excluded papers published only as abstracts or presented in conferences without full publication and papers published in languages other than English.

## Data extraction

We extracted data on study design, population characteristics (age, gender, diagnosis), associated symptoms, and treatment. We recorded dose and length of time using the medication or substance prior to the withdrawal event. When a dose range was given, a mean dose was calculated and used. We recorded the length of time from discontinuation until onset of catatonia. We recorded whether each episode of catatonia was associated with psychotic or autonomic symptoms. We also recorded treatments used, whether or not they were effective, and if so, the time to response.

## Results

The electronic search strategy outlined in the methods section identified 46 papers that met inclusion and exclusion criteria. These 46 papers were made up of case series and case reports that together reported on 55 cases of withdrawal catatonia. The cases described eight different types of medications causing withdrawal catatonia, including withdrawal catatonia related to benzodiazepines (24 cases), clozapine (20 cases), combined alcohol and benzodiazepines (4 cases), alcohol (2 cases), glutethimide (2 cases), zolpidem (1 case), gabapentin (1 case), and gamma-hydroxybutyric acid (1 case). Full details of the benzodiazepine and clozapine withdrawal catatonia cases are outlined in Tables 1 and 2.

**Table 1:** Published reports of benzodiazepine withdrawal catatonia

Study	Age	Gender	Psychiatric/neurologic diagnoses	Benzodiazepine use	Diazepam equivalents (per 24 h)	Duration of use	Time until symptoms	Psychotic symptoms	Autonomic symptoms	Successful treatment	Time to response	Scale Used for Diagnosis	DSM-V Criteria Met
Hauser et al.^32^	29	M	Complex partial seizures	Clorazepate 45 mg daily	30 mg	Not specified	3 days	Yes (hallucinations)	No	Diazepam, clorazepate	15 min	No	Yes
	30	F	Complex partial seizures, depression	Clonazepam 4 mg daily	80 mg	Not specified	3 days	Yes (hallucinations, delusions)	No	Lorazepam	Immidiate	No	Yes
Rapport and Covington^33^	70	F	Not specified	Diazepam 30 mg daily, alprazolam (dose unknown)	30 mg (for diazepam)	Diazepam: 4 years alprazolam: 6 months	7 days	Yes (hallucinations, delusions)	No	Lorazepam	“Minutes”	No	Yes
Rosebush and Mazurek^34^	88	M	Nil	Clonazepam 1.5 mg daily	30 mg	15 years	5 days	Yes (hallucinations)	Yes (tachycardia)	Lorazepam	45 min	No	Yes
	70	M	Major depressive disorder with psychosis	Alprazolam 2 mg daily	40 mg	6 months	4 days	No	Yes (elevated blood pressure)	Lorazepam	1 h	No	Yes
	66	F	Bipolar disorder, alcohol abuse, benzodiazepine abuse	Oxazepam (dosage unknown), temazepam (dosage unknown), lorazepam 4 mg daily (started for a 2-week course)	40 mg (for lorazepam)	10 years	7 days after lorazepam decrease from 4 mg to 1 mg daily	No	Yes (elevated blood pressure, tachycardia, diaphoresis)	Lorazepam	30 min	No	No
	53	F	Nil	Diazepam 40−60 mg daily	40−60 mg (mean = 50 mg)	15 years	2 days	No	No	Lorazepam	Not specified	No	Yes
	63	F	Major depressive disorder, anxiety disorder	Diazepam 30 mg daily	30 mg	20 years	3 days	No	No	Lorazepam	1 h	No	Yes
Carroll^35^	42	F	Major depressive disorder, panic disorder	Lorazepam 1 mg TID (taking up to 7 mg daily), meprobamate 400 mg TID (taking up to 2400 mg daily)	30−70 mg (for lorazepam) (mean = 50 mg)	Not specified	7 days	Yes (hallucinations)	No	Lorazepam, diazepam	Not specified	No	Yes
Glover et al.^36^	62	M	Schizophrenia	Lorazepam 0.5 mg BID	10 mg	Not specified	2 days	Active psychosis prior to benzodiazepam discontinuation and onset of catatonia	No	Lorazepam	2 h	No	Yes
Deuschle and Lederbogen^37^	51	M	Chronic fatigue and insomnia	Bromazepam 18 mg daily	30−36 mg (mean = 33 mg)	9 years	5 days	Yes (hallucinations, delusions)	No	Lorazepam	4 h	No	Yes
Carroll et al.^38^	61	M	Anxiety disorder	Diazepam 5 mg TID	15 mg	Not specified	Not specified	Yes (delusions)	Yes (elevated blood pressure, tachycardia)	Lorazepam	1 h	Yes (BFCRS)	Yes
Brown and Freeman^39^	60	M	Post-traumatic stress disorder, major depressive disorder, anxiety disorder—not otherwise specified	Clonazepam 2 mg TID	120 mg	Not specified	7 days	No	Yes (fever, elevated blood pressure, tachycardia, diaphoresis)	Lorazepam, clonazepam	“Immediate”	No	Yes
Lauterbach et al.^40^	39	M	Neurosyphilis	Temazepam 30 mg daily, lorazepam 1−3 mg PRN	15 mg (for temazepam), 10−30 mg (for lorazepam) (mean = 20 mg)	Temazepam: 34 days, lorazepam: 3 days	5 days after temazepam discontinuation, 2 days after last lorazepam dose	Active psychosis prior to benzodiazepam discontinuation and onset of catatonia	No	Lorazepam	48 h	No	Yes
Carroll et al.^41^	64	F	Dysthymia	Lorazepam 5 mg daily	50 mg	6 months	“Immediately”	No	No	Lorazepam	“Hours”	No	No
Parameswaran et al.^42^	73	F	Nil	Temazepam 40 mg daily	20 mg	40 years	4 days after dose decrease to 20 mg	No	No	Midazolam	“Rapid”	No	Yes
Amos^43^	59	F	Depression, anxiety	Lorazepam, 4 mg daily	40 mg	Not specified	3 days	No	Yes (fever, tachycardia)	Lorazepam	5 min	Yes (BFCRS)	Yes
Wang et al.^44^	39	F	Schizophrenia	Lorazepam 2 mg daily	20 mg	Not specified	2 days	Yes (hallucinations, delusions)	No	Lorazepam	Not specified	Yes (BFCRS)	Yes
Sivakumar et al.^45^	57	M	Depression	Lorazepam 2 mg daily	20 mg	7 years	2 days	No	No	Lorazepam	Not specified	Yes (BFCRS)	Yes
Saddawi-Konefka et al.^46^	78	F	Depression, anxiety	Alprazolam 0.5 mg TID	30 mg	40 years	4 days	No	No	Lorazepam	15 min	No	Yes
	77	F	Depression, anxiety, panic disorder	Chlordiazepoxide (dose unknown)	Dose unknown	15 years+	3 days	No	No	Lorazepam	1 day	No	Yes
Holoyda and Xiong^47^	31	M	Insomnia, anxiety	Alprazolam 3 mg daily	60 mg	2 years	3 days	Yes (hallucinations, thought disorder)	Yes (elevated blood pressure)	Lorazepam	15 min	Yes (BFCRS)	Yes
Oldham and Desan^28^	62	F	Major depressive disorder, unspecified anxiety disorder, minor neurocognitive disorder, fibromyalgia	Clonazepam 1 mg daily	20 mg	2 years	8 days	No	No	Clonazepam	“Hours”	Yes (BFCRS)	Yes
Peng et al.^48^	61	F	Anxiety	Lorazepam 2 mg daily	20 mg	Not specified	3 days	No	Yes (elevated blood pressure, tachycardia)	Midazolam, lorazepam	“Minutes”	No	No

**Table 2:** Published reports of clozapine withdrawal catatonia

Study	Age	Gender	Psychiatric/neurologic diagnoses	Clozapine dose prior to discontinuation	Duration of use	Time until symptoms	Psychotic symptoms	Autonomic symptoms	Succesful treatment	Time to response	Unsuccesful treatment	Scale used for diagnosis	DSM-V criteria met
Parsa et al.^49^	25	M	Schizophrenia	225 mg	5 months	7 days	Yes (hallucinations)	No	Melperone (partial), loxapine (partial)	6 weeks (but never returning to baseline)	Fluphenazine, ECT, haloperidol	No	No
Lee and Robertson^50^	30	M	Schizophrenia	350 mg	14 months	36 h	Yes (thought disorder)	Yes (diaphoresis, fever, elevated blood pressure, tachycardia, tachypnea)	Clozapine	3 weeks	Pimozide, haloperidol, chlorpromazine, clonazepam, benztropine, lorazepam	No	Yes
Yeh et al.^51^	55	M	Schizophrenia	400 mg	6 years	<7 days	Yes (hallucinations)	Yes (diaphoresis, flushed face, bradycardia and tachycardia)	Clozapine, trihexyphenidyl	7 days	x	Yes (BFCRS)	Yes
Hung et al.^52^	42	F	Schizophrenia	500 mg	Not specified	14 days	Yes (hallucinations, delusions)	No	Clozapine	Not specified	Lorazepam, diazepam, ECT	No	No
Kalogeropoulou et al.^53^	37	F	Schizophrenia	350 mg	10 years	<7 days	Yes (disorganized speech and behavior)	Yes (fever)	Clozapine	Not specified	Lorazepam, risperidone, amisulpride	No	Yes
Bastiampillai et al.^29^	58	F	Schizoaffective disorder	150 mg	4 years	3 days	No	Yes (fever, diaphoresis, fluctuating blood pressure)	ECT	10 ECT sessions	Lorazepam	No	Yes
Thanasan and Jambunathan^54^	“Middle aged”	M	Schizoaffective disorder	200 mg	5 years	7 days	Yes (hallucinations, delusions)	Yes (fever, fluctuating blood pressure, tachycardia)	Bromocriptine, “anticholinergic”, diazepam	12 days	x	No	No
Wadekar and Syed^55^	49	F	Schizophrenia	550 mg	“Many years”	5 days	No	No	Clozapine	2 days	Lorazepam, benazepril, olanzapine	Yes (BFCRS)	No
Dhillon et al.^56^	61	F	Schizoaffective disorder	400 mg	16 years	Not specified	Yes (positive symptoms)	No	Aripiprazole, ECT	Not specified	x	No	No
Kanagasundram and Chengappa^57^	45	F	Schizophrenia	400 mg	7 years	“Rapidly (within days)”	Yes (hallucinations, delusions)	Yes (fever)	Amisulpride	2 days	Haloperidol	No	Yes
Kumar et al.^58^	29	M	Schizophrenia	250 mg	3 months	2 days	No	No	Lorazepam, ECT	4 ECT treatments	x	No	Yes
Cerit et al.^59^	46	M	Schizophrenia	200 mg	10 years	5 days	Yes (hallucinations, delusions)	Yes (fever, tachycardia)	Clozapine, lorazepam	2 days	ECT	Yes (BFCRS)	No
Wang et al.^44^	39	F	Schizophrenia	200 mg	8 years	“Immediate”	Yes (hallucinations, delusions)	No	Clozapine	Not specified	x	Yes (BFCRS)	Yes
Erol et al.^60^	27	M	Schizophrenia	150 mg	4 years	5 days	Yes (hallucinations, delusions)	Yes (fever, tachycardia, labile blood pressure, diaphoresis)	ECT	1 ECT treatment	Diazepam	No	Yes
Koch et al.^61^	33	F	Schizophrenia	250 mg	6 months	14 days	Yes (hallucinations)	Yes (fever)	Olanzapine, lorazapam, ECT	10 ECT sessions, 4 weeks	Quetiapine	No	Yes
Ariyasinghe and Abeyasinghe^62^	44	F	Schizophrenia	Not specified	10 years	“Recently” after discontinuation	No	No	Clozapine	1 week	x	No	Yes
Saddawi-Konefka et al.^46^	48	M	Schizoaffective disorder	Not specified	Not specified	7 days	No	No	ECT	8 ECT treatments	Lorazepam	No	Yes
Koychev et al.^63^	22	M	Psychotic Illness	300 mg	5 weeks	4 days	No	No	Lorazepam	7 days (rigidity improved in 45 min)	x	No	No
Ingole et al.^64^	44	M	Schizoaffective disorder	200 mg	Not specified	7 days	No	Yes (autonomic instability)	Lorazepam	3 days	x	Yes (BFCRS)	Yes
Bilbily et al.^30^	38	M	Schizophrenia	400 mg	7 years	10 days	No	No	Lorazepam, Clozapine	3 months	Haloperidol, Diphenhydramine	No	Yes

### Benzodiazepine withdrawal catatonia

Eighteen articles^28,32–48^ described 24 cases of benzodiazepine withdrawal catatonia (Table 1). The average patient age was 58 years (range 29−88 years) and the male to female ratio was 1:1.4. The use of benzodiazepines in terms of diazepam equivalents ranged from 10 to 120 mg daily. The mean daily dose was 38 mg and the median dose was 30 mg. The duration of use, when stated, ranged from 34 days to 40 years, with a median of 9 years. The onset of catatonia following last benzodiazepine dose ranged from 2 to 8 days, and occurred in 7 days or less in all but 1 case. Psychotic symptoms were associated with 11 of the cases (46%) and autonomic symptoms were associated with 8 of the cases (33%). Successful treatment of the catatonic episode was achieved with re-initiation of a benzodiazepine in all 24 cases. The time to response ranged from 15 min to 4 h, with two outlier cases indicating a time to response of 1 and 2 days respectively.

Of the 24 case reports of benzodiazepine withdrawal catatonia, confidence of catatonia being the diagnosis of the withdrawal event was high in 21 of the cases based on the specific symptoms described in the reports being sufficient to meet DSM-V criteria for catatonia. Of those 21 cases, 6 reported the use of the Bush Francis Catatonia Rating Scale (BFCRS) to make the diagnosis. The confidence of a catatonia diagnosis was lower in three cases that did not describe any or enough specific symptoms in their report to verify a DSM-V diagnosis of catatonia and also did not report the use of a rating scale in making the diagnosis.

### Clozapine withdrawal catatonia

Twenty articles^29,30,44,46,49–64^ described 20 cases of clozapine withdrawal catatonia (Table 2). The average patient age was 41 years old (range 22−61 years) and the male to female ratio was 1.22:1. The mean daily dose of clozapine used prior to the catatonic event was 304 mg, with a range of 150−550 mg. The median dose was 275 mg. The duration of use ranged from 5 weeks to 16 years, with a median of 5.5 years. The onset of catatonia following last clozapine dose ranged from 36 h to 14 days, and occurred in 7 days or less in all but three cases. Psychotic symptoms were associated with 12 of the cases (60%) and autonomic symptoms were associated with 10 of the cases (50%). Various treatments were found to be unsuccessful in the treatment of clozapine withdrawal catatonia, including benzodiazepines in six of the cases and ECT in three of the cases. Successful treatment was observed with a variety of treatments, the two most common being re-initiation of clozapine (9 cases) and the use of ECT (6 cases). Time to response ranged from 2 days to 3 months.

Confidence of catatonia being the diagnosis of the withdrawal event was high in 15 of the 20 cases based on enough specific symptoms being described to meet DSM-V criteria for catatonia or the BFCRS being used to make the diagnosis. The confidence of a catatonia diagnosis was lower in the five cases that did not describe any or enough symptoms to verify a DSM-V diagnosis of catatonia and also did not report use of a rating scale in making the diagnosis.

### Other withdrawal catatonia

Two cases of alcohol withdrawal catatonia^65,66^ and four cases of mixed alcohol and benzodiazepine withdrawal catatonia^28,67–69^ were identified. In addition, two cases of catatonia on withdrawal from glutethimide^70,71^, and single cases of catatonia on withdrawal from gabapentin^72^, zolpidem^73^, and gamma-hydroxybutyric acid^74^ were identified.

One case report described a patient who experienced catatonia on withdrawal of clozapine and later on withdrawal of benzodiazepines independent of each other^44^. It was therefore included in both sections of the results.

## Discussion

This is the first review examining the entire spectrum of withdrawal catatonia. One main finding of our review was that the two primary medications associated with withdrawal catatonia were clozapine and the medication class of benzodiazepines. Clozapine and benzodiazepines are both known to cause withdrawal symptoms. In the case of clozapine, recognized withdrawal symptoms include rebound psychosis^75^ and rebound movement disorders including dystonias and dyskinesias^76,77^. In the case of benzodiazepines, withdrawal symptoms commonly include rebound anxiety and insomnia^27^.

This principal finding brings up the question of whether or not there may be a shared pharmacological mechanism between clozapine and benzodiazepines that underlie both these medications being associated with withdrawal catatonia. Clozapine and benzodiazepines have the structural similarity of both being based around a diazepine rings. Clozapine is in the dibenzodiazepine class, which refers to it having two benzene rings fused to a diazepine ring^78^. Benzodiazepines contain a single benzene ring fused to a diazepine ring^79^. There are significant structural differences between the two as well however, and the clinical significance of the structural similarities is unclear.

Drug withdrawal or discontinuation symptoms have been suggested to be a result of the pharmacological profile of a drug, its neurobiological targets and the adaptations of the body to these targets following its use^80^. When a drug is discontinued, and eliminated from the system, the persistence of the adapted state in the absence of the drug leads to withdrawal symptoms^81^. Therefore clozapine and benzodiazepines may share a common neurobiological target leading to the receptor changes that result in a catatonic state. We hypothesize that a commonality possibly involves the GABA system. All the medications and psychoactive substances identified to cause withdrawal catatonia have an effect on increasing activity in the GABA system. Benzodiazepines facilitate GABA activity through allosteric modulation of GABA_A_ receptors^82^. Clozapine increases GABA levels through effects on different receptors located on GABA interneurons^83^ and through acting as an agonist at GABA_B_ receptors, the evidence for which is reviewed later in the article. All of the other identified compounds in our review including alcohol, glutethimide, gabapentin, zolpidem, and GHB have been associated with increasing GABA activity as well^84–88^. Of note, there were no cases of ECT withdrawal catatonia identified, despite ECT being a treatment for catatonia that also increases GABA activity^7,8^. This may be due to ECT having longer lasting effects, limiting the sudden fluctuation of GABA activity required for withdrawal symptoms to occur.

Another key finding of our review was that clozapine was the only antipsychotic reported to have caused withdrawal catatonia within a 2-week period following discontinuation. This finding has to be viewed in the context of clozapine making up only a relatively small proportion of all antipsychotics prescribed in the community. This brings up the question of what are the unique aspects of clozapine’s pharmacology that result in it causing withdrawal catatonia when no other antipsychotic does. We hypothesize that one aspect may be clozapine’s possible effect on the GABA system, a receptor system that is not typically associated with the action of other antipsychotic medications. Clozapine has been clearly demonstrated to have superior efficacy than other antipsychotics in treatment-resistant schizophrenia^89^. The reasons for this are not well understood and therefore identification of unique aspects of clozapine’s pharmacology is important.

GABA activity is likely a key factor in the clinical picture of withdrawal catatonia but it would be too simplistic to view it as the only receptor system involved. While one should refrain from making conclusions from a review consisting solely of case reports, an examination of the characteristics of the catatonic episodes in the cases suggests that benzodiazepine and clozapine withdrawal catatonia are phenotypically different from one another. While increasing GABA activity may be a shared feature of clozapine and benzodiazepine activity, there are many differing aspects of their pharmacology and these differences likely underlie the observed phenotypic variations. This is further explored as we discuss proposed etiological mechanisms for each.

### Benzodiazepine withdrawal catatonia

In the 24 cases of benzodiazepine withdrawal catatonia, the benzodiazepine dose was substantial and the length of use was typically long term. There were no withdrawal catatonic episodes documented after very short-term regular use or intermittent as-needed use, despite the fact that benzodiazepines are frequently used in this manner. We hypothesize that this is because in order to create an environment in which withdrawal catatonia could occur, chronic use of benzodiazepines is required to create compensatory receptor changes. Chronic potentiation of activity at GABA_A_ receptors would likely result in downregulation of GABA_A_ receptor function^90,91^. When the GABA promoting drugs are then suddenly withdrawn from the downregulated GABA_A_ receptors, a state of GABA deficiency would result, leading to catatonia. Reinstitution of benzodiazepines, which act as GABA_A_ agonists, would be expected to have a high treatment response rate through correction of the GABA deficiency. This was indeed the clinical observation, as all 24 cases of benzodiazepine withdrawal catatonia responded rapidly to treatment with benzodiazepines.

Symptoms of psychosis accompanied the catatonic symptoms in about half of the cases. The psychotic symptoms most commonly reported were hallucinations with or without delusions. These symptoms are not core features of catatonia. This finding is somewhat surprising as only two of the cases reported pre-existing psychotic symptoms and therefore the psychotic symptoms that occurred were new-onset and could not be attributed to pre-existing illness in most cases. One possible explanation is that there is a component of delirium in benzodiazepine withdrawal catatonia. Alcohol, which also acts on GABA_A_ receptors, is associated with a withdrawal delirium with prevalent hallucinations. It has been previously suggested that catatonia associated with benzodiazepine withdrawal and withdrawal delirium may exist along a spectrum with a convergence in pathophysiology^28^. In the cases we identified, eight described disorientation, which is a central feature of delirium. Disorientation or other features of delirium may have been present in other cases as well; however, assessing mental status in the presence of a condition that commonly features mutism is very difficult and may not be possible to do. An alternative explanation is that when a state of GABA hypoactivity occurs with benzodiazepine withdrawal, a hyper-dopaminergic state is stimulated and this could result in the emergence of psychotic symptoms. Downregualtion of GABA function has been understood to increase dopaminergic activity in the ventral tegmental area through disinhibition of pyramidal neurons^92^.

### Clozapine withdrawal catatonia

In the 20 cases of clozapine withdrawal catatonia, the dose of clozapine used was within the average dose range used for schizophrenia and the length of time on treatment was typically long term with a median time of 5.5 years. As was the case with benzodiazepine withdrawal, this represents a significant period of regular daily use, likely needed to create an environment capable of causing withdrawal effects through receptor adaptations. Which receptors undergo changes with long-term clozapine use is less clear than with benzodiazepine use, due to clozapine’s complex multireceptor mechanism of action.

The most striking difference between benzodiazepine and clozapine withdrawal catatonia was the response to treatment. In contrast to the 100% rate of response to benzodiazepines in the cases of benzodiazepine withdrawal catatonia, clozapine withdrawal catatonia showed a poor response to benzodiazepines. In six of the cases benzodiazepines were initially trialed and found to be ineffective. An additional seven of the 20 cases trialed a benzodiazepine and reported it as being effective; however, in five of those cases the benzodiazepine was used as an adjunct to other treatments and therefore it is difficult to determine how much benefit was due to the benzodiazepine itself. There were only two reports of a benzodiazepine being an effective treatment when used in isolation for clozapine withdrawal catatonia. The treatment that was found to be most effective for clozapine withdrawal catatonia was reinstitution of clozapine, which successfully treated the catatonic symptoms in all of the nine cases it was trialed. This included multiple cases in which clozapine was re-instituted after failed trials of benzodiazepines and ECT. The second most effective treatment was ECT, which was successful in treating six cases both on its own as well as an adjunct to medication. There were however three cases that found ECT ineffective in treating the catatonic episode that subsequently responded to clozapine. This ECT failure rate is somewhat high considering ECT has been found to be effective in treating 80−100% of all forms of catatonia^93^. The time to response for the treatment of clozapine withdrawal catatonia was variable, but in general it was a much longer time period than that seen with benzodiazepine withdrawal catatonia. When using benzodiazepines to treat benzodiazepine withdrawal catatonia, a response was often seen after a single dose; however, when using clozapine to treat clozapine withdrawal catatonia, a response was not typically seen until multiple doses of clozapine were administered over a number of days or weeks. We hypothesize that a delay in the initiation of treatment with clozapine and use of a slow titration schedule may have contributed to the delay in response. In many cases of clozapine withdrawal catatonia, clozapine was also not re-instituted until several other treatments had failed.

The finding that clozapine withdrawal catatonia was frequently not responsive to treatment with benzodiazepines and had only a mixed response to ECT highlights the complexity of the pharmacology of clozapine and likely suggests contribution of non-GABA components to the etiology of clozapine withdrawal catatonia. The other phenotypic differences between benzodiazepine and clozapine withdrawal catatonia (Table 3) are also likely related to clozapine’s effect on multiple neurotransmitter systems associated with catatonia and the resultant complex receptor adaptations. This includes the GABA, dopamine, and acetylcholine systems and their complex interactions.

**Table 3:** Differences in the clinical presentation of benzodiazepine withdrawal catatonia and clozapine withdrawal catatonia

	Benzodiazepine withdrawal catatonia	Clozapine withdrawal catatonia
Dose of treatment	Average-high (benzodiazepine dose)	Average (clozapine dose)
Duration of treatment	Long term	Long term
Accompanying psychotic symptoms	Prevalent	Very prevalent
Response rate to treatment with benzodiazepines	High	Low
Most effective treatments	Benzodiazepines	Clozapine, ECT
Time to response	Minutes to hours	Days to weeks

### Clozapine and GABA

Clozapine’s association with catatonia, a condition thought to be due to GABA hypoactivity, adds some clinical support to pharmacokinetic and genetic studies that have demonstrated clozapine to increase GABA activity. Studies in rats have found that the vesicular GABA transporter (VGAT), the protein responsible for transfer of GABA from cytoplasm to synaptic vesicles, is upregulated by clozapine^94^. Other findings have suggested that epigenetic downregulation of the expression of several GABAergic genes due to gene promoter hypermethylation is associated with psychotic symptoms in schizophrenia and bipolar disorder. Clozapine has been found to induce DNA-demethylation of the GABA gene promoters, thus potentially correcting deficiencies in the GABA system resulting in a reduction of psychotic symptoms^95^. Cases of polysomnograph-confirmed rebound insomnia after clozapine withdrawal have been described where the specific effects on sleep are similar to those seen after benzodiazepine discontinuation^96,97^. This is suggestive of the sleep disturbance in clozapine discontinuation being possibly related to alterations in GABA activity.

Clozapine has been shown to have different effects on GABA levels in different areas of the brain. Studies on rats have found that clozapine increased levels of GABA in the hippocampus and ventral tegmental area, had minimal effects on GABA levels in the medial prefrontal cortex, and decreased GABA levels in areas of the striatum^83^. Clozapine’s effect on GABA levels is thought to be due to its blockade of multiple receptors located on GABA interneurons^98^. For instance, clozapine’s effect on increasing temporal lobe GABA levels is thought to involve its antagonism of D2, D4, and α_2_ adrenoreceptors located on the GABA interneurons of that region^83^.

With clozapine increasing GABA levels in certain areas of the brain, GABA receptor downregulation occurring after long-term clozapine use is a theoretical possibility. If receptor downregulation occurs and clozapine is then discontinued, a state of GABA hypoactivity resulting in a catatonic episode may occur. Supporting this theory is a finding that the level of GABA in rat brains decreased compared to controls beginning 3−6 days after clozapine was abruptly discontinued^83^. This finding was region-specific with the most significant decrease being observed in the ventral tegmental area, but was also observed in the dorsal hippocampus, nucleus accumbens, and globus pallidus.

Clozapine also has direct effects on GABA receptors, the clinical significance of which is unclear. Evidence from binding studies support clozapine having antagonist activity at GABA_A_ receptors^99^; however, the effects are weak. Evidence from human and animal studies on neuronal activity have also suggested that clozapine increases activity at GABA_B_ receptors^100–102^. Clozapine use could therefore theoretically result in downregulation of GABA_B_ receptors; however, evidence does not support an association between GABA_B_ hypoactivity and catatonia. There are no reported cases of baclofen, a GABA_B_ agonist, being effective in treating catatonia and furthermore, two case reports have described baclofen-induced catatonia^103,104^, suggesting a possible association between catatonia and GABA_B_ hyperactivity. The findings in relation to GABA_B_ activity are based on very limited evidence and further experimental evidence would be needed to clarify any association between catatonia and GABA_B_ activity.

Clozapine withdrawal catatonia did not respond well to treatment with benzodiazepines. This is likely because clozapine’s primary action on the GABA system is not at the GABA receptor level where benzodiazepines exert their effect, but rather through clozapine’s effects on various receptors located on GABA interneurons. It is also likely that multiple receptor systems are involved in clozapine withdrawal catatonia and that just addressing GABA hypoactivity may not be sufficient in resolving the clinical syndrome.

### Clozapine and dopamine

Neuroleptic-induced catatonia is thought to be due to the blockade of D2 dopamine receptors by antipsychotics creating a hypodopaminergic state^105^. Neuroleptic-induced catatonia has mostly been attributed to high potency typical antipsychotics but has been reported with atypical antipsychotics as well^12,13^. Clozapine however has been successfully used for treating catatonia rather than inducing it. It has consistently been shown in PET studies to have low D2 receptor occupancy^106^ and looser D2 binding compared to most other antipsychotics^107^. In line with this is the finding that while antipsychotics worsen motor symptoms in Parkinson’s disease through D2 blockade in the nigrostriatal pathway, clozapine is effective in treating psychotic symptoms in Parkinson’s disease without worsening motor symptoms^108^.

The finding that clozapine treats catatonia while other antipsychotics can induce it suggests that clozapine’s dopaminergic activity may be more complex than just D2 antagonism. A catatonic state could hypothetically occur on withdrawal of an agent that increases dopaminergic transmission if compensatory downregulation of dopamine receptors occurred. Interestingly, there is evidence that clozapine has partial dopamine agonist activity. In a rat model it was found that clozapine induced hypothermia through dopamine stimulation and this effect was fully antagonized by a D1 receptor antagonist, suggesting that clozapine has agonist activity at D1 receptors^109^. Evidence from other animal models support clozapine having partial agonist activity at D2 receptors^110,111^. Another mechanism in which clozapine has been shown to increase dopamine levels is through its effects on serotonin receptors. Clozapine’s antagonism of 5-HT_2A_ receptors and activation of 5-HT_1A_ receptors have been found to enhance dopamine release in the prefrontal cortex^112^. The potential that clozapine may increase prefrontal cortex dopamine levels may explain its anticatatonic potential.

Catatonia has similarities in its presentation to extrapyramidal side effects of medication due to D2 blockade. Specifically, severe parkinsonism could look similar to the stuporous form of catatonia. Therefore, it could be postulated that the cases we identified in our review as withdrawal catatonia were instead manifestations of severe parkinsonism. While we acknowledge this as a possibility, we do not believe this to be the case for a few reasons. First, most of the cases described symptoms that met DSM-V criteria for a diagnosis of catatonia, and most of those symptoms would not be classified as an extrapyramidal side effect. Second, classic parkinsonian symptoms such as cogwheel rigidity, pill-rolling tremor, masked facies, or shuffling gait were not described in any of the cases we reviewed and none reported use of a scale for extrapyramidal symptoms. The exception to this is bradykinesia, which in its most severe form would overlap with the stupor seen in catatonia. Third, unlike typical antipsychotics, clozapine has minimal association with causing parkinsonism or worsening the motor symptoms of Parkinson’s disease. Finally, we conducted a search of clozapine withdrawal movement disorders which revealed cases of clozapine withdrawal dyskinesias and dystonias^77^, but no cases of clozapine withdrawal parkinsonism.

### Clozapine and other receptors

Clozapine has extremely potent anticholinergic activity, considered to be comparable to that of atropine^113^. Sudden withdrawal of anticholinergic medication results in rebound overactivity of the cholinergic system. While cholinergic hyperactivity may not have a direct role in causing catatonia, it has been associated with causing autonomic disturbance^114^ and psychotic symptoms^115^, both of which were frequently observed in the cases of clozapine withdrawal catatonia.

### Withdrawal catatonia versus illness relapse

An argument could be made that the occurrence of catatonia following clozapine discontinuation within 14 days could be related to a relapse of the underlying illness rather than withdrawal effects from the medication. All patients identified in this group had a diagnosis of a primary psychotic illness, which can also present with catatonic features. There are a number of reasons however that we believe the catatonia in these cases to be related to withdrawal rather than illness relapse. First, the cases of withdrawal catatonia that we identified all occurred within 14 days of clozapine discontinuation, and all but three of those occurred within 7 days or less of discontinuation. This time frame supports a mechanism related to discontinuation as it matches the time frame in which somatic discontinuation symptoms are seen upon stopping clozapine^114^. This is also the time frame that is seen for the occurrence of a rapid onset psychosis that can follow clozapine discontinuation, a process that is also believed to be distinct from a relapse of the underlying illness^75^. Second, of the 20 cases identified, only eight had experienced a previous episode of catatonia and in five of those cases, the previous episodes of catatonia had only occurred also immediately following clozapine discontinuation. Therefore, only 3 of the 20 cases had previously experienced a catatonic episode that had been unrelated to clozapine discontinuation. Based on this, it is our view that the cases of catatonia identified were less likely to be due to a relapse of the underlying illness considering that in the majority of the cases (85%), catatonia unrelated to clozapine withdrawal had not previously been a specific feature of the patient’s illness presentation.

### Treatment guidelines for withdrawal catatonia

The 24 cases of benzodiazepine withdrawal catatonia identified presented in a nearly identical fashion to “typical” cases of catatonia previously described in the literature in regard to both symptoms and treatment response. We would therefore suggest following pre-established guidelines for the treatment of catatonia when encountering a case of benzodiazepine withdrawal catatonia, including using lorazepam as the medication of choice^116^. The use of antipsychotic medications with high D2 receptor antagonism, such as typical antipsychotics, should be avoided. If lorazepam is not effective or if the patient is experiencing high fevers or is medically compromised, ECT should be considered^116^. Conservative measures such as ensuring adequate hydration and being mindful of falls risk due to the use of sedating medication are essential as well.

As clozapine withdrawal catatonia has not been widely studied in the past, treatment suggestions are based on the accumulation of data from this literature review. Based on our findings, we would suggest the first-line treatment for clozapine withdrawal catatonia be early recognition and reinstitution of clozapine. Clinical guidelines recommend that when more than 2 days of clozapine treatment have been missed, clozapine should be re-started at a dose of 12.5−25 mg and increased slowly by 25−50 mg daily over 1−2 weeks until a therapeutic dose is reached^117^. The purpose of the slow taper is to minimize the occurrence of adverse effects including orthostatic hypotension and seizures. There is however emerging evidence that rapid titration of clozapine using a higher starting dose and increasing by up to 100 mg daily is safe and provides faster resolution of psychotic symptoms^118^. We speculate that an early and fast titration of clozapine would also result in a reduction of the duration of symptoms in clozapine withdrawal catatonia. A rapid inpatient titration with close monitoring should therefore be considered in cases where there are no pre-existing conditions that predispose an individual to experience adverse effects, such as old age, cardiac disease, or seizure disorder.

In addition to reinstitution of clozapine, the use of adjunct benzodiazepines can be considered, but are unlikely to be effective if used as a monotherapy. Sometimes reinstitution of clozapine is not possible due to various factors including drug intolerance or clozapine-induced agranulocytosis. When clozapine reinstitution is not possible, we would suggest the use of ECT. Consideration of conservative measures such as those described above are even more important in the treatment of clozapine withdrawal catatonia due to its association with autonomic instability and its prolonged course.

## Study limitations

There are several important limitations to our review. All the studies included were either case reports or case series, which by their nature are subject to overinterpretation, publication bias, and recall bias^119^. Additionally, this type of research does not allow for inference of epidemiological measures such as incidence or prevalence. There was a lack of consistency in reporting relevant clinical information among the case reports, making comparison difficult at times. Clinical descriptions varied from extensive reporting on symptoms and use of catatonia rating scales to cases with a catatonia diagnosis made by the authors but with minimal specific symptomology described. In fact, few cases reported the use of a rating scale such as the BFCRS and instead relied on a clinical diagnosis. When symptoms were not explicitly stated and no rating scale was used, our confidence in the stated diagnosis of catatonia being the true diagnosis was lowered. While all reports of catatonia included met our set criteria and were interpreted categorically as being related to medication withdrawal, it is possible that there were other causative factors for the catatonic episode that went unreported.

It is also possible that there was underreporting of withdrawal catatonia cases unrelated to clozapine or benzodiazepine use.

## Further research and conclusions

We conducted a comprehensive review on the phenomenon of withdrawal catatonia with a goal of identifying all medications and psychoactive substances associated with it. Clozapine and benzodiazepines were the principal agents identified. With the prevailing view that catatonia is associated with hypoactivity of the GABA system, our findings are suggestive of GABA activity being a potential pharmacological similarity between clozapine and benzodiazepines. Benzodiazepines have relatively straightforward involvement with the GABA system, acting as allosteric agonists at GABA_A_ receptors. Clozapine however has a much more complex mechanism involving action on multiple receptor systems that interface with GABA interneurons resulting in region-specific changes in serum GABA levels. Clozapine also has complex interactions with the dopamine and acetylcholine systems which have also been implicated in the pathophysiology of catatonia and its associated symptoms.

For withdrawal catatonia to occur, several years of treatment are usually required, suggesting that receptor adaptations are an important etiological component for developing withdrawal catatonia. Long-term benzodiazepine use could result in GABA_A_ receptor downregulation. When the benzodiazepine is then abruptly discontinued, a GABA-deficient state could result in predisposing an individual to develop catatonia. Long-term clozapine use could lead to downregulation of the multiple receptors clozapine acts on that have complex and indirect interactions with the GABA system. When clozapine is then abruptly discontinued, this could also result in a GABA-deficient state leading to the development of a catatonic episode. It is the complex multireceptor effects of clozapine that explains why clozapine withdrawal catatonia was more severe and responded mainly to reinstitution with clozapine rather than benzodiazepines.

Clozapine is an antipsychotic medication of significant interest due to its unique efficacy in treatment-resistant schizophrenia. The aspects of clozapine’s pharmacology that underlie this unique feature are yet to be fully established. Our finding was that clozapine was the only antipsychotic to have caused withdrawal catatonia within 14 days of discontinuation. One possible reason for this is that clozapine’s uniqueness as an antipsychotic is related to its effect on the GABA system. We therefore speculate that this unique aspect may also underlie clozapine’s superior efficacy in treatment-resistant schizophrenia. Abnormalities in the GABA system have been demonstrated in patients with schizophrenia. It is possible that treatment-resistant schizophrenia is related to problems with GABA transmission that is addressed by clozapine. Further research into clozapine and its possible interaction with the GABA system is warranted, as this could have a significant impact on the development of more novel and targeted pharmaceuticals.
